# Effect of game-based high-intensity interval training program on the executive function of children with ADHD: Protocol of a randomized controlled trial

**DOI:** 10.1371/journal.pone.0272121

**Published:** 2022-07-28

**Authors:** Fenghua Sun, Gary Chi-Ching Chow, Clare Chung-Wah Yu, Ying-Fung Ho, Duo Liu, Stephen Heung-Sang Wong, Parco Ming-Fai Siu, Simon B. Cooper, David Jenkins

**Affiliations:** 1 Department of Health and Physical Education, The Education University of Hong Kong, Hong Kong SAR, China; 2 Department of Rehabilitation Sciences, The Hong Kong Polytechnic University, Hong Kong SAR, China; 3 Department of Special Education and Counselling, The Education University of Hong Kong, Hong Kong SAR, China; 4 Department of Sports Science and Physical Education, The Chinese University of Hong Kong, Hong Kong SAR, China; 5 Division of Kinesiology, School of Public Health, The University of Hong Kong, Hong Kong SAR, China; 6 School of Science & Technology, Nottingham Trent University, Nottingham, United Kingdom; 7 School of Health and Behavioural Sciences, University of the Sunshine Coast, Sippy Downs, QLD, Australia; Public Library of Science, UNITED KINGDOM

## Abstract

**Background:**

Attention-deficit/hyperactivity disorder (ADHD) is a common developmental disorder in childhood, with a 5%-6% worldwide prevalence. Children with ADHD often demonstrate impaired executive function, which is closely related to the development of the commonly observed behavioral problems such as inattention, impaired inhibition, and hyperactivity. The purpose of this study is to examine whether a game-based high-intensity interval training (HIIT) program can improve the executive function of children with ADHD, compared with a traditional structured aerobic exercise program and a non-treatment control group.

**Methods/Design:**

A total of 42 children with ADHD will be recruited to participate in this three-arm school-based randomized controlled trial. An 8-week specially designed game-based HIIT (GameHIIT) program and a traditional game-based structured aerobic exercise (GameSAE) program will be delivered to those children randomly assigned to these two intervention groups, while the children in the control group will maintain their regular physical activity over the same period. A number of outcome measures including executive function, cerebral hemodynamic response, physical activity, physical fitness, and enjoyment and adherence to the intervention will be assessed for both groups at baseline (T0), immediately after the intervention period (T1), and after the follow-up period (T2).

**Discussion:**

HIIT has recently emerged as a feasible and efficacious strategy for increasing physical health outcomes and cognitive function, including executive function, in healthy young people. However, research has yet to investigate whether the executive function of children with ADHD can be effectively enhanced through HIIT. If, as hypothesized, GameHIIT program improves outcomes for children with ADHD, the present research will inform the development of targeted exercise programs that can be more broadly used with this particular population.

## Background

There is strong evidence that regular physical activity (PA) is associated with a range of physical health benefits for school-age youth, including improvements in body composition, physical capacity, and overall health-related indicators (e.g., blood pressure, insulin resistance, lipid profile) [1]. Emerging evidence also suggests that PA and physical fitness have a positive effect on mental health [2], cognitive function and academic performance [3]. Executive functions are generally defined as “high-level cognitive processes” that manage other basic cognitive functions [4]. They consist of functions such as planning, self-regulation, initiation and inhibition, and cognitive flexibility [5, 6]. These functions are believed to be important prerequisites for successful learning in preadolescent children [7], predict better health and wealth, and has been associated with a reduced likelihood of being convicted of a criminal offence [8]. Several recent meta-analyses have suggested that PA may positively affect cognition and executive function in children [9, 10]. Despite the known benefits of an active lifestyle, more than half of Hong Kong’s children and young people fail to follow the current physical activity recommendations [11], and trends show a decline in health-related physical fitness [12]. Executive functions develop from early childhood and through adolescence into adulthood [13, 14], with large developmental changes occurring during the elementary school years [15, 16]. Accordingly, effective interventions implemented early in life when the higher cortex is still developing, have the potential to elicit significant long-term improvements [17]. Therefore, it is important to find effective strategies to promote the PA of children, so as to improve their executive function during their childhood.

Attention-deficit/hyperactivity disorder (ADHD) is a commonly diagnosed developmental disorder, with a 5.3% worldwide prevalence in children and adolescents [18]. The most common symptoms include inattention, impulsivity, impaired inhibition, and hyperactivity [19]. Also, young people with ADHD are often characterized by dysfunction in high-level cognitive functions such as executive function [20]. This executive dysfunction may play an important role in the commonly observed behavioral problems in children with ADHD. There are a number of ways to treat ADHD, e.g., medication, psychotherapy, psychoeducation, neuro- feedback, behavior management, etc. Of these treatments, PA and exercise have emerged as effective strategies to manage ADHD given that neither are associated with negative side effects (see recent reviews [21–24]). To summarize, although not always consistently, PA, especially moderate- to high-intensity aerobic exercise, may improve the emotion/mood, behavior, executive function, and some physical measures of children with ADHD. Acute aerobic exercise may have a positive effect on a variety of measures with a large effect size of up to 1.26 in children with ADHD [23]. Two studies have reported medium-to-large effects of acute exercise on executive function [25, 26]. Long-term exercise interventions have also been shown to benefit children with ADHD, with improvements in some measures showing a large effect size up to 0.96 [23]. However, these benefits have been mainly described as an improvement of behavioral and emotional problems [22]. Several studies have reported that long-term ‘mixed exercise programs’ may have moderate to significant effects on several aspects of executive function (e.g., inhibition) and attention in children and adolescents with ADHD [27–29], but other studies did not report the same findings [30]. Therefore, so far evidence-based suggestions regarding the optimal exercise for children with ADHD remain somewhat inconsistent [22].

Research into the relationship between PA and ADHD has generally involved mixed exercise programs at a low- to moderate-intensity; running and stationery cycling have been the most common exercise modes. Whether structured PA is effective for young people is yet to be examined [31]. Children’s habitual PA patterns are characterized by participation in games or “unpredictable” sports activities (e.g., football, basketball) [32]. Given that children’s intrinsic motivation, or level of enjoyment, is also a strong predictor of PA participation [33], any intervention program should be designed to optimize their enjoyment of PA and thus enhance the likelihood of long-term adherence. When compared to structured exercise such as running or cycling, games-based PA arguably provides a more attractive, acceptable, sustainable and enjoyable exercise model for young children [34]. To date, only a few studies have examined the effect of an acute bout of team game-based activity on cognitive function in healthy children and adolescents [35–38]. Research has found that different aspects of cognitive function, such as free recall memory, attention, executive function, and working memory are improved following an acute bout of game-based team exercise (e.g., basketball, tennis) [35–38]. For children with ADHD, mixed exercise protocols have generally been used in intervention programs, and only a few studies have used team sport-based games as part of their exercise intervention [29, 39]. However, given the design of these studies, it is not possible to distinguish the sole effect of game-based PA from those of the mixed exercise protocols in these studies. A recent study has reported that a 12-week table tennis exercise has positive effects on the gross motor skills and some of the executive function performances (mainly inhibition) in the ADHD training group, compared with the ADHD non-training group and a control group [40]. It should be noted that the game-based PA in these aforementioned studies was generally ‘aerobic’ in nature, and completed at low- to moderate-intensities.

Recently high-intensity interval training (HIIT) has emerged as a feasible and efficacious strategy for improving the physical health of young people [41, 42]. The HIIT can be completed in a short period of time, while resulting in equivalent physiological adaptations to longer sessions of traditional aerobic training [43]. Recent research has suggested that traditional HIIT intervention programs, including running and cycling, may improve executive function in healthy children and adolescents [44–46]. It has been suggested that a very brief HIIT intervention over two weeks reduced off-task behavior and enhanced selective attention in primary school children [44, 45]. For children with ADHD, to the best of our knowledge, only one recent study [47] was conducted to investigate the effect of a traditional HIIT program on physical fitness, motor skills, social behavior, and quality of life. In this randomized controlled trial, 28 boys with ADHD were assigned to either a traditional HIIT group or a standard multimodal therapy (TRAD) group. After the three-week intervention, the authors reported that HIIT was more effective in improving motor skills, self-esteem, relations with friends, competence, and subjective ratings of attention, compared with TRAD. However, despite this encouraging preliminary evidence, it remains unclear whether HIIT can be adopted to treat children with ADHD to improve their executive function, a key aspect in many facets of life.

While the limited findings regarding the effects of HIIT on executive function are encouraging, and HIIT has emerged as an enjoyable and effective exercise for children [42], previous studies have tended to prescribe HIIT interventions with a focus on running and jumping [44–46]. The effect of game-based HIIT interventions on the executive function of children has yet to be investigated. Research of this nature is needed, particularly to determine whether game-based HIIT can improve outcomes (e.g., executive function, social behavior, sports skills, etc.) for children with ADHD. Therefore, the aim of the proposed study is to investigate the effect of two different kinds of exercise programs, i.e., an 8-week game-based HIIT (GameHIIT) program and an 8-week game-based structured aerobic exercise (GameSAE) program, on the executive function of children with ADHD. The hypothesis of the proposed study is that both GameHIIT and GameSAE programs will significantly improve the executive function of children with ADHD, compared with those in the control group. A secondary hypothesis is that the GameHIIT group may confer additional benefits when compared with the GameSAE group.

## Methodology

### Participants

A total of 42 children with ADHD will be recruited from local schools. The inclusion criteria are: (1) Chinese children aged 6–13 years; (2) a clinical diagnosis of ADHD by developmental pediatricians or clinical psychologists/psychiatrists; (3) a physician/psychologist’s recommendation for participation. The exclusion criteria are: (1) diagnosed with a major neurodevelopmental or psychiatric disorder (e.g., autism spectrum disorder, intellectual disability.); (2) acute/chronic diseases that may affect engagement in physical activity; and (3) a tendency to experience convulsions. Informed consent will be obtained from the school principal, parents, and study participants before the study begins. Human research ethics approval has been sought from the Human Research Ethics Committee of the University (Ref. no. A2018-2019-0098).

### Sample size calculation

The sample size is calculated using G*Power 3.1. To elucidate the differences in the executive function tests with a statistical power of 0.9, a conservative effect size of 0.65 based on a previous systematic review with the average effect size calculated regarding the effect of exercise on executive function in children with ADHD [24], a two-tailed alpha level of 0.05, it is determined that 10 participants per group will provide adequate power to detect statistically significant differences. Assuming a 30% loss in the intervention, we will need to approach about 42 eligible participants to achieve the planned sample. The protocol has been registered at ClinicalTrials.gov (Identifier: NCT05308758).

### Study design

**Fig 1:**
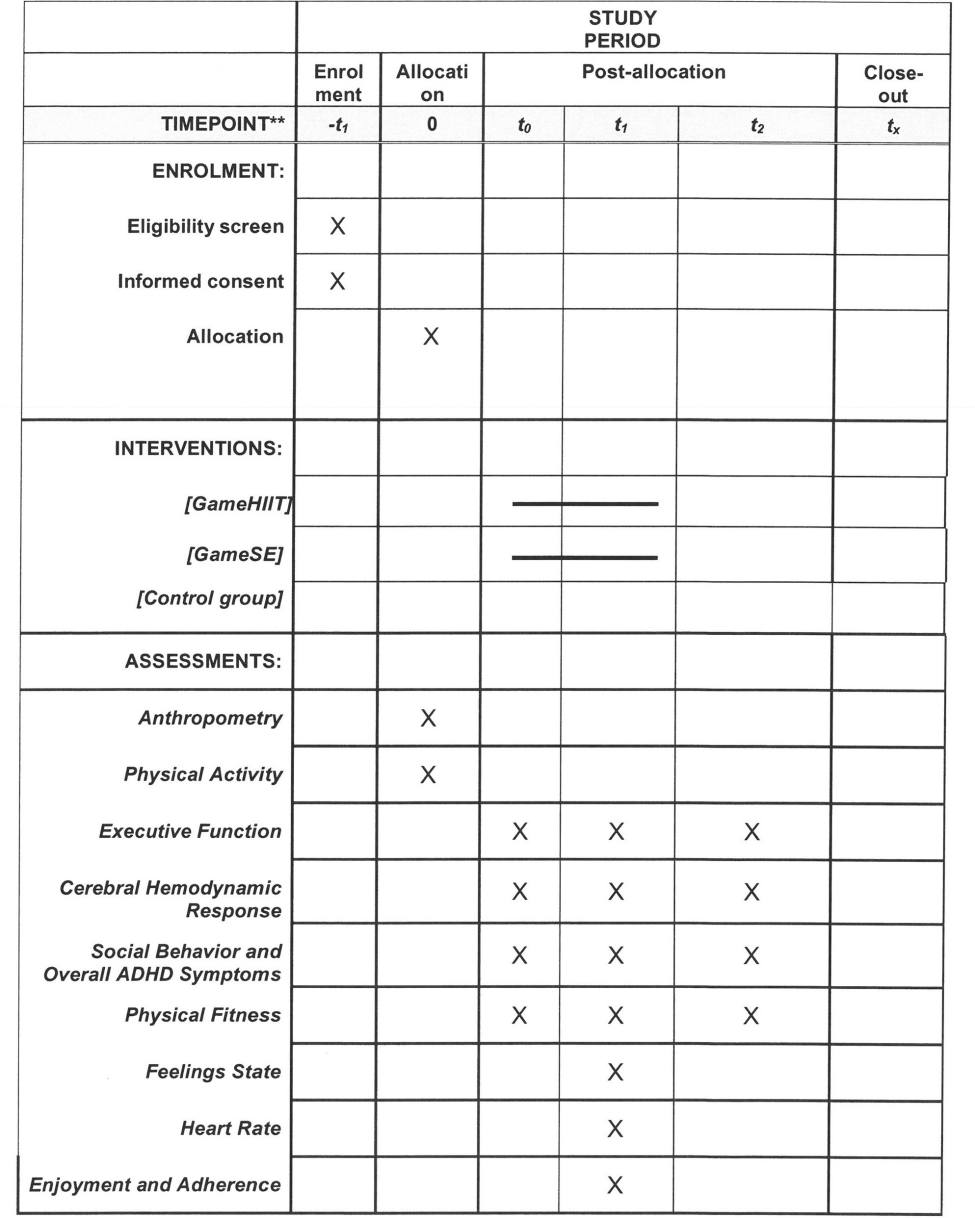
The schedule of enrolment, interventions, and assessments.

**Fig 2:**
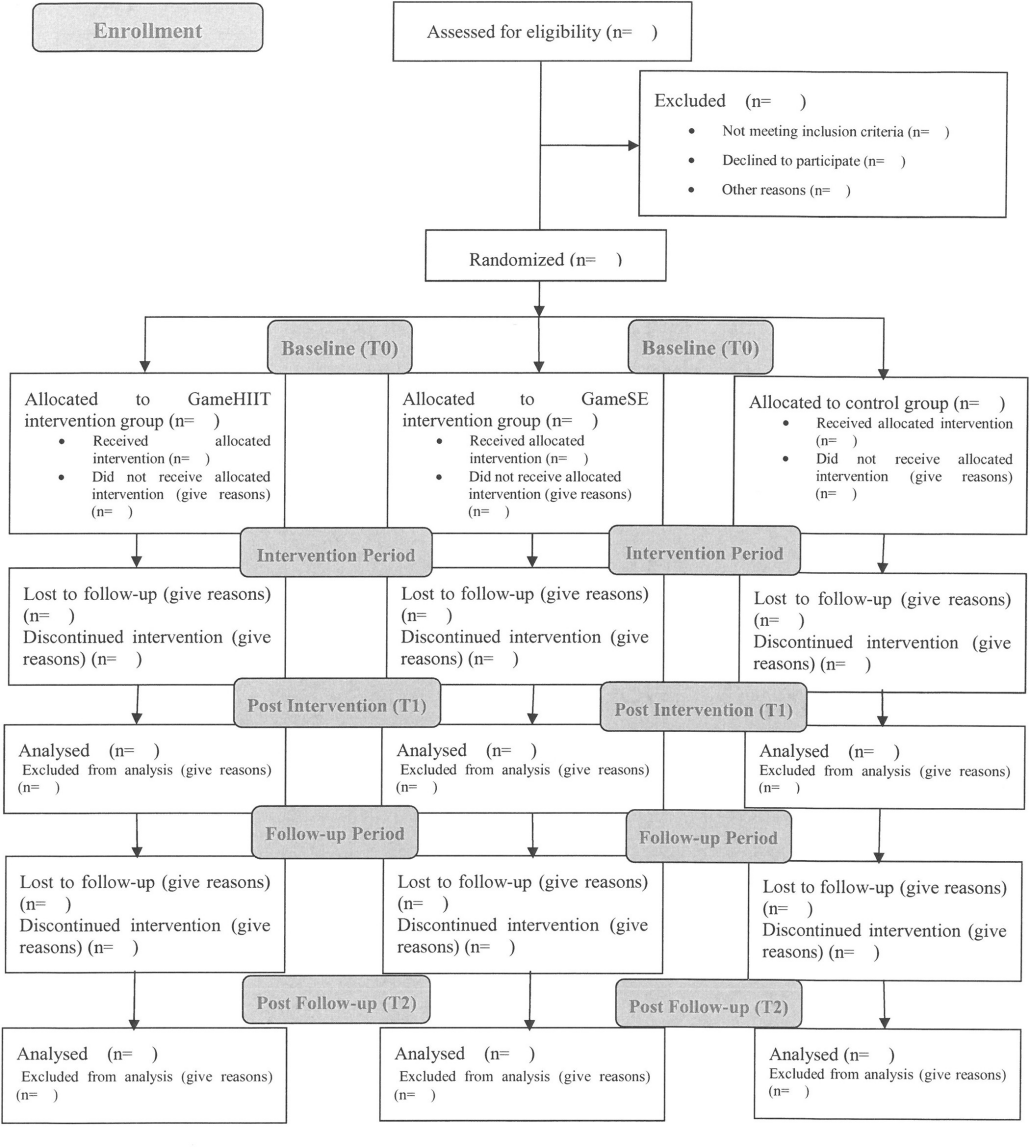
Flow diagram of the study.

A three-arm school-based randomized controlled trial (RCT) will be conducted to evaluate the effects of two different kinds of 8-week training programs on the executive function of children with ADHD. The design, conduct, and reporting for the RCT adhere to the guidelines of the Consolidated Standards of Reporting trials (http://www.consort-statement.org/) [48]. Participants will be randomly assigned to the GameHIIT group, the GameSAE group, or a non-treatment control group using a random number-producing algorithm (with a 1:1:1 allocation ratio within each school). A stratified random sampling procedure will be conducted that considers gender, IQ, and medication status. Equal numbers of boys and girls with similar general intelligence will be included in the three groups. Children taking medication will be equally distributed to the three groups. Participants will not be blinded to treatment allocation because of the intervention nature. To avoid contamination between treatment groups, intervention deliverers will be provided with a list of students in the intervention program. Only those on the list can participate in the intervention. During the 8-week intervention period, participants in the control group will maintain their regular PA levels. For ethical reasons, a waiting list control study design will be adopted. That is, participants in the control group will receive either GameHIIT intervention or GameSAE intervention according to their preference after an 8-week formal intervention period. Accordingly, participants in the other two groups will have another 8-week follow-up. Therefore, it is expected that all participants in the three groups will benefit from the proposed study. The SPIRIT schedule of enrolment is shown in Fig 1, and the flow diagram of the study design is shown in Fig 2.

### Intervention protocols

In the GameHIIT group, a specially designed game-based training program with HIIT in nature will be delivered to the participants for 8 weeks. A small-sided games (SSGs) approach in rugby will be adopted in this intervention program as it is effective in developing physical and technical capabilities in children [49], and provides similar physical stimulus, regardless of the experience of the children [50]. Importantly, rugby has been introduced in a large scale to primary and secondary schools in Hong Kong. There will be two training sessions each week. In each training session, there will be four sets of training programs separated by 3 minutes of passive recovery in accordance with a previous study [51]. Each set of activities will last for around 5 minutes; therefore, the total duration of each training session will be approximately 30 minutes. A small group size (4–6 children per group) will be adopted to facilitate individual supervision and adaption of the exercise program. Also, certain social, cognitive and coordinative elements will be included which may play an important role in liking the neuropsychological concept of executive function. All the training programs will be organized for participants after school hours. A qualified rugby coach will be hired to implement the rugby training program. To encourage maintenance of an appropriate level of exercise intensity, participants will be fitted with heart rate monitors (Polar H7), which will be connected to a central iPad application (Polar Team). The coach will be able to view real-time HR data during training. If necessary, we will adjust the exercise intensity to ensure that HR can reach the target HR zone. Adherence to the designed GameHIIT protocol will be recorded by the coach in each training session.

In the GameSAE group, participants will attend a tailor-made game-based exercise training program [52]. Similar to GameHIIT, the intervention will comprise 8 weeks of structured aerobic exercise sessions, lasting one hour on average in each session and up to twice per week. Six to eight stations of multidimensional exercises will be set up for each session. Adopting the train-the-trainer (TTT) model, training will be provided by front-line healthcare providers or trained helpers. Children will be instructed to finish the exercises in all stations one after another in a predetermined order. The exercise program has 3 stages and each of the stages last around four weeks. In the first stage, the aim is to build trust with their coaches, and paired group activities are included. In the second stage, the exercise intensity will be increased to promote cardiopulmonary endurance and muscular strength. In the final stage, the exercise intensity for each session will be higher than that of the previous stages and there will be large group activities as well. To record the progress of the training classes and provide feedback to the front-line healthcare providers, a professional coach and research assistant will take part in the training once every two weeks.

### Outcomes measurements

Before (T0) and after (T1) the 8-week intervention period, as well as another 8-week follow-up (T2), several different indicators will be recorded, including executive function, cerebral hemodynamic response, weekly PA levels, physical fitness, feeling state, and enjoyment and adherence to the intervention. All assessments will be conducted by trained research staff blinded to group allocation. To ensure the accuracy and consistency of the measurements, a measurement training session and protocol manual, including specific instructions for conducting all assessments, will be provided to the research staff. A senior researcher will be present during all the testing sessions. All physical assessments will be conducted in a sensitive manner (e.g., weight/waist circumference will be measured in a private setting), and the cognitive function tests and questionnaires will be completed under exam-like conditions. Also, participants will be instructed to follow similar diets on the main trial days. Only distilled water will be allowed before the tests in the main trials.

### Primary outcome

#### Executive function

Executive function will be assessed using a battery of tests on a laptop computer that will take approximately five minutes to complete. The battery of tests includes the Colour-Word Stroop Test (CWST), Corsi Block Tapping Test (CBTT), Wisconsin Card Sorting Test (WCST), and Tower of London Test (TLT) which are classic tasks that measure inhibition response, one of the important components of executive function. Previous studies have reported a medium to a large effect size of the different exercise intervention programs on the inhibition of children and adolescents with ADHD [27, 28, 40]. In these two tests, both reaction time and response accuracy will be recorded and analyzed. This battery has been used previously by research group members to investigate the effect of exercise on cognition in young people [53]. The instructions for each test will be provided to the participants and they will be allowed to ask questions for clarification. Participants will be familiarized with the testing battery seven days prior to the main trial, and each test will start with 3–6 practice stimuli to re-familiarize participants with the task at hand and eliminate any potential learning effects. In the main trial, participants will complete the tests individually. They must remain silent from the other participants so that they cannot interact with others while taking the tests.

The instructions for each test will be provided to the participants and they will be allowed to ask questions for clarification. Participants will be familiarized with the testing battery seven days prior to the main trial, and each test will start with 3–6 practice stimuli to re-familiarize participants with the task at hand and eliminate any potential learning effects. In the main trial, participants will complete the tests independently so that they would not be disturbed by others while taking the tests.

Besides computer tests, the Behavior Rating Inventory of Executive Function (second Edition) will also be used to supplement the executive function tests.

#### Cerebral hemodynamic response

Accompanied by the executive function test, the cortical hemodynamic response in the prefrontal cortex will also be recorded using a multi-channel fNIRS (Octamon fNIRS system, Artinis, Netherland) applying two wavelengths of near-infrared light (785 and 830 nm). The device consists of eight light sources and two detectors secured onto a head cap. The device will be placed over the left and right prefrontal cortex according to the guidelines in the handbook provided by the manufacturer. The data will be analyzed as described [54].

### Secondary outcomes

#### Anthropometry

Body height, weight, as well as waist and hip circumference will be measured three times. All measurements will follow the Anthropometry Procedures Manual of National Health and Nutrition Examination Survey (NHANES).

#### Social behavior and overall ADHD symptoms

Conners’ Teacher Rating Scale 15-Item (CTRS-15) [55] will be used to measure the social behaviors of participants. It has been widely used to assess problematic behaviors in children with ADHD. The Overall ADHD symptoms will be assessed by the Attention- Deficit/Hyperactivity-symptoms and Normal-behaviors (SWAN) rating scale [56].

#### Physical activity

Children’s leisure-time PA will be determined using both an accelerometer (ActiGraph, Shalimar, USA) and a validated and modified version of the Physical Activity Questionnaire for Children (PAQ-C) [57]. Participants will be required to wear an accelerometer on their right hip for seven days to collect objective data of PA levels. The time on and time off of wearing the accelerometer each day will be recorded, and the data will be used to estimate the time spent in moderate-to-vigorous PA (MVPA). The PAQ-C is a 7-day self-report questionnaire designed to assess daily activities from moderate to vigorous range, and the score is in a continuous range from 1 (low active) to 5 (high active).

#### Feelings state

A one-item Feelings State questionnaire will be administrated before and after each intervention session (total 24 sessions). Participants will be asked to respond on an 11-point scale (-5 = very bad to +5 = very good) to the question *How are you feeling right now*? Mean pre- and post-workout scores will be calculated for each session [58].

#### Heart rate

Participants will be fitted with Polar H7 heart rate monitors during the training sessions, which will be connected to a central iPad application. The mean heart rate for the entire session and the mean maximum heart rate will be tracked over the study period.

#### Enjoyment and adherence

Enjoyment will be assessed by the Physical Activity Enjoyment Scale, which is a valid and reliable tool for evaluating perceived enjoyment [59]. Adherence to the intervention program will be evaluated by attendance frequency and dropout rate.

#### Physical fitness

Physical fitness (cardiovascular fitness, muscular strength, and speed-agility) will be assessed using the ALPHA fitness test battery [60]. Briefly, cardiovascular fitness will be assessed by the 20 m shuttle run test; muscular strength will be assessed by the handgrip strength test and standing long jump test; and speed-agility will be assessed by the 4 × 10 m shuttle run test [60].

### Statistical analyses

Statistical analyses of the primary and secondary outcomes will be conducted with the IBM SPSS Statistic for Windows, Version 20.0 (2010 SPSS Inc., IBM Company Armonk, NY). Intervention effects for the primary and secondary outcomes will be examined by two-way (trial × time) analysis covariance (ANCOVA), including group as a fixed factor, pre-post intervention difference (change) as the dependent variable, and age, attendance, sports skill, and ADHD symptoms as covariates. Effect sizes (ES) will be presented as partial eta squared values (*ŋ*^2^). Pairwise comparison will be performed (post-hoc) with Bonferroni correction, with ES presented as Cohen’s *d*. All data will be presented as mean ± SD, and significance will be set as *p* < 0.05 for all data analyses.

## Discussion

A limited number of studies have investigated the acute and chronic effects of different types of exercise interventions on children with ADHD. In general, there is evidence showing the acute positive effects of exercise on executive function. As for chronic effects, however, decreases in emotional and behavioral problems have been reported. Several studies have found that long-term exercise may have medium to large effects on inhibition and attention in children with ADHD. However, usually mixed exercise programs with low- to moderate-intensity aerobic nature were used in these studies, and running and cycling were two common exercise modes used. HIIT has recently emerged as feasible and efficacious for increasing physical health outcomes, including the executive performance of children and adolescents. These benefits may extend to children with ADHD. Although a traditional HIIT program can be completed in a short period while providing physiological adaptations equivalent to those provided by longer sessions of traditional aerobic training, it generally involves shuttle runs or cycling, activities that many children are not likely to enjoy. This may lead to their subsequent disinterest in the program. In contrast to running or cycling, team games closely resemble the habitual physical activity patterns of children and so they may find these activities more acceptable and enjoyable. In previous studies, some game-based activities have been used as part of mixed exercise programs to promote the physical/mental health of children with ADHD. However, most of these game-based activities are actually low- to moderate-intensity exercise in nature. Therefore, if the demands of HIIT are embedded in team games, children’s enjoyment of, and adherence to completing, an effective exercise program could be potentially increased. Importantly, this would integrate the benefits of both game-based activities and HIIT.

There are several strengths of this study. Firstly, this will be the first randomized controlled trial to investigate the potential benefits of a game-based HIIT program on the executive function of children. Such an intervention program is especially important for children with ADHD as it involves not only physical exercise, but also social behavior and sports skills. These will be quite helpful in attenuating some typical behavioral problems in children with ADHD. Secondly, the largest developmental changes in executive function occur from early childhood, through adolescence, into adulthood. Accordingly, implementing effective interventions in the early stages of life will potentially maximize their impact. Thirdly, this study combines the strengths of HIIT and game-based activities. As both activities are shown to improve the executive function of children, it is expected that the combined beneficial effect will be even stronger. Finally, the potential mechanism behind the beneficial effects of game- based HIIT on executive function will also be explored. As improvement in executive function has been suggested to be related to physiological changes in prefrontal oxygenation (e.g., higher oxyhaemoglobin), non-invasive measurement obtained via fNIRS will be used in the proposed study to monitor the cerebral hemodynamic response as participants perform the cognitive tasks.

In conclusion, this study attempts to investigate the effect of an 8-week game-based HIIT program on the executive function of children with ADHD. It is expected that the findings of the proposed study will contribute to the literature in this impactful and novel area, as well as inform the development of specific exercise programs targeted at children with ADHD.

## Supporting information

S1 Checklist SPIRIT 2013 checklist: Recommended items to address in a clinical trial protocol and related documents*. (DOC)

S1 Protocol (PDF)
