# Supplementary material for: Effect of game-based high-intensity interval training program on the executive function of children with ADHD: Protocol of a randomized controlled trial
Source: PLoS One. 2022 Jul 28;17(7):e0272121. doi: 10.1371/journal.pone.0272121 (PMC9333304; doi:10.1371/journal.pone.0272121)
Supplement: S1 Checklist — (DOC) [file pone.0272121.s001.doc]

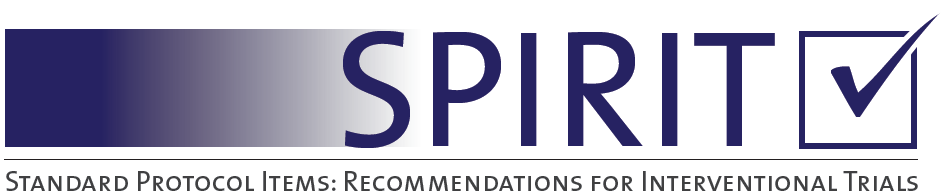


SPIRIT 2013 Checklist: Recommended items to address in a clinical trial protocol and related documents*

| Section/item | ItemNo | Description |  | |
| --- | --- | --- | --- | --- |
| **Administrative information** | | |  | |
| Title | 1 | Effect of Game-based High-Intensity Interval Training Program on the Executive Function of Children with ADHD: Protocol of A Randomized Controlled Trial |  | |
| Trial registration | 2a | Protocol Registration and Results System, ClinicalTrials.gov ID: NCT05308758 |  | |
|  | 2b | Included. |  | |
| Protocol version | 3 | 20220409, Fenghua SUN |  | |
| Funding | 4 | This study was substantially supported by a grant from the Research Grants Council of the Hong Kong Special Administrative Region, China (Project No. EdUHK 18603120). |  | |
| Roles and responsibilities | 5a | Feng-Hua Sun, Department of Health and Physical Education, The Education University of Hong Kong, Hong Kong SAR, China  Gary Chi-Ching CHOW, Department of Health and Physical Education, The Education University of Hong Kong, Hong Kong SAR, China  Clare Chung-Wah YU, Department of Rehabilitation Sciences, The Hong Kong Polytechnic University, Hong Kong SAR, China  Ying-Fung HO, Department of Health and Physical Education, The Education University of Hong Kong, Hong Kong SAR, China  Duo LIU, Department of Special Education and Counselling, The Education University of Hong Kong, Hong Kong SAR, China  Stephen Heung-Sang WONG, Department of Sports Science and Physical Education, The Chinese University of Hong Kong, Hong Kong SAR, China  Parco Ming-Fai SIU, Division of Kinesiology, School of Public Health, The University of Hong Kong, Hong Kong SAR, China  Simon B. COOPER, School of Science & Technology, Nottingham Trent University, United Kingdom  David JENKINS, School of Health and Behavioural Sciences, University of the Sunshine Coast, Australia |  | |
|  | 5b | **Trial Sponsor**: Research Grants Council of the Hong Kong Special  Administrative Region, China (Project No. EdUHK 18603120)  **Sponsor’s Reference:** The Education University of Hong Kong, Hong Kong SAR, China  **Contact name**: Fenghua SUN  **Address:** Rm D4-2F-25, Department of Health and Physical Education, The Education University of Hong Kong, Tai Po, Hong Kong.  **Tel:** 852-29487854.  **Fax:** 852-29487848.  **Email:** fhsun@eduhk.hk. |  | |
|  | 5c | This funding source had no role in the design of this study and will not have any role during its execution, analyses, interpretation of the data, or decision to submit results. |  | |
|  | 5d | NA |  | |
| Introduction |  |  |  | |
| Background and rationale | 6a | **Background**  Introduction: Attention-deficit/hyperactivity disorder (ADHD) is a common developmental disorder in childhood, with a 5%-6% worldwide prevalence. Children with ADHD often demonstrate impaired executive function, which is closely related to the development of the commonly observed behavioural problems such as inattention, impaired inhibition, and hyperactivity.  Mechanisms: Among the current treatments, PA and exercise have emerged as effective strategies to manage ADHD given that neither are associated with negative side effects (see recent reviews [1-4]).PA, especially moderate- to high-intensity aerobic exercise, may improve the emotion/mood, behaviour, executive function, and some physical measures of children with ADHD. Acute aerobic exercise may have a positive effect on a variety of measures with a large effect size up to 1.26 in children with ADHD [3]. High-intensity interval training (HIIT) has recently emerged as a feasible and efficacious strategy for increasing physical health outcomes and cognitive function, including executive function, in healthy young people [5, 6].  Existing knowledge: Several recent meta-analyses have suggested that PA may positively affect cognition and executive function in children [7, 8]. Recent research has suggested that traditional HIIT intervention programs, including running and cycling, may improve executive function in healthy children and adolescents [9-11]. It has been suggested that a very brief HIIT intervention over two weeks reduced off-task behavior and enhanced selective attention in primary school children [9, 10]. For children with ADHD, to the best of our knowledge, only one recent study [12] was conducted to investigate the effect of a traditional HIIT program on physical fitness, motor skills, social behavior, and quality of life. In this randomized controlled trial, 28 boys with ADHD were assigned to either a traditional HIIT group or a standard multimodal therapy (TRAD) group. After the three-week intervention, the authors reported that HIIT was more effective in improving motor skills, self-esteem, relations with friends, competence, and subjective ratings of attention, compared with TRAD. However, despite this encouraging preliminary evidence, it remains unclear whether HIIT can be adopted to treat children with ADHD to improve their executive function, a key aspect in many facets of life.  Need for a trial: The limited findings regarding the effects of HIIT on executive function are encouraging, and HIIT has emerged as an enjoyable and effective exercise for children [6], previous studies have tended to prescribe HIIT interventions with a focus on running and jumping [9-11]. The effect of game based HIIT interventions on the executive function of children have yet to be investigated. Research of this nature is needed, particularly to determine whether game based HIIT can improve outcomes (e.g., executive function, social behavior, sports skills, etc.) for children with ADHD. |  | |
|  |  |  |  | |
|  | 6b | An 8-week specially designed game-based HIIT (GameHIIT) program and a traditional game-based structured aerobic exercise (GameSAE) program will be delivered to those children randomly assigned to these two intervention groups, while the children in the control group will maintain their regular physical activity over the same period. |  | |
| Explanation for choice of comparators | 7 | The hypothesis is that both GameHIIT and GameSAE programs will significantly improve the executive function of children with ADHD, compared with those in the control group. A secondary hypothesis is that the GameHIIT group may confer additional benefits when compared with the GameSAE group. |  | |
| Objectives | 8 | A three-arm school-based randomized controlled trial (RCT) will be conducted to evaluate the effects of two different kinds of 8-week training programs on the executive function of children with ADHD. |  | |
| Trial design | 8 | A three-arm school-based randomized controlled trial (RCT) will be conducted to evaluate the effects of two different kinds of 8-week training programs on the executive function of children with ADHD. |  | |
| Methods: Participants, interventions, and outcomes | | | 9 | Local school. |
| Study setting | 10 | The inclusion criteria are: (1) Chinese children aged 6-13 years; (2) a clinical diagnosis of ADHD by developmental paediatricians or clinical psychologists/psychiatrists; (3) a physician/psychologist’s recommendation for participation. The exclusion criteria are: (1) diagnosed with a major neurodevelopmental or psychiatric disorder (e.g., autism spectrum disorder, intellectual disability.); (2) acute/chronic diseases that may affect engagement in physical activity; and (3) a tendency to experience convulsions. |  | |
| Eligibility criteria | 11a | The design, conduct, and reporting for the RCT adheres to the guidelines of the Consolidated Standards of Reporting trials (http://www.consort-statement.org/) [13].  An 8-week specially designed game-based HIIT (GameHIIT) program and a traditional game-based structured aerobic exercise (GameSAE) program will be delivered to those children randomly assigned to these two intervention groups, while the children in the control group will maintain their regular physical activity over the same period. A number of outcome measures including executive function, cerebral hemodynamic response, physical activity, physical fitness, and enjoyment and adherence to the intervention will be assessed for both groups at baseline (T0), immediately after the intervention period (T1), and after the follow-up period (T2). |  | |
| Interventions | 11b | During the whole trials or intervention, participants may get exhausted according to the physical activity intensity, if the participants had chest pain, or lose your balance because of dizziness, or have a bone or muscle pain, the participants would be recommended to stop the intervention. |  | |
|  | 11c | To encourage maintenance of an appropriate level of exercise intensity, participants will be fitted with heart rate monitors (Polar H7), which will be connected to a central iPad application (Polar Team). The coach will be able to view real-time HR data during training. If necessary, we will adjust the exercise intensity to ensure that HR can reach the target HR zone. Adherence to the designed GameHIIT protocol will be recorded by the coach in each training session. |  | |
|  | 11d | Children taking medication will be equally distributed to the three groups. Participants will not be blinded to treatment allocation because of the intervention nature. To avoid contamination between treatment groups, intervention deliverers will be provided with a list of students in the intervention program. Only those on the list can participate in the intervention. |  | |
|  | 12 | **Primary Outcome**  Executive Function  Executive function will be assessed using a battery of tests on a laptop computer that will take approximately five minutes to complete. The battery of tests includes the Colour-Word Stroop Test (CWST), Corsi Block Tapping Test (CBTT), Wisconsin Card Sorting Test (WCST), and Tower of London Test (TLT) which are classic tasks that measure inhibition response, one of important components of executive function. Previous studies have reported medium to large effect size of the different exercise intervention programs on inhibition of children and adolescents with ADHD [14-16]. In these two tests, both reaction time and response accuracy will be recorded and analysed. This battery has been used previously by research group members to investigate the effect of exercise on cognition in young people [17].  Cerebral Hemodynamic Response  Accompanied by the executive function test, the cortical hemodynamic response in the prefrontal cortex will also be recorded using a multi-channel fNIRS (Octamon fNIRS system, Artinis, Netherland) applying two wavelengths of near-infrared light (785 and 830 nm). The device consists of eight light sources and two detectors secured onto a head cap. The device will be placed over the left and right prefrontal cortex according to the guidelines in the handbook provided by the manufacturer. The data will be analyzed as described [18].  **Secondary Outcomes**  Anthropometry  Body height, weight, as well as waist and hip circumference will be measured three times. All measurements will follow the Anthropometry Procedures Manual of National Health and Nutrition Examination Survey (NHANES).  Social Behavior and Overall ADHD Symptoms  Conners' Teacher Rating Scale 15-Item (CTRS-15) [19] will be used to measure the social behaviors of participants. It has been widely used to assess problematic behaviors in children with ADHD. The Overall ADHD symptoms will be assessed by the Attention-Deficit/Hyperactivity-symptoms and Normal-behaviors (SWAN) rating scale [20].  Physical Activity  Children’s leisure-time PA will be determined using both an accelerometer (ActiGraph, Shalimar, USA) and a validated and modified version of the Physical Activity Questionnaire for Children (PAQ-C) [21]. Participants will be required to wear an accelerometer on their right hip for seven days to collect objective data of PA levels. The time on and time off wearing the accelerometer each day will be recorded, and the data will be used to estimate the time spent in moderate-to-vigorous PA (MVPA). |  | |
| Outcomes |  | The PAQ-C is a 7-day self-report questionnaire designed to assess daily activities from moderate to vigorous range, and the score is in a continuous range from 1 (low active) to 5 (high active).  Feelings State  A one-item Feelings State questionnaire will be administrated before and after each intervention session (total 24 sessions). Participants will be asked to respond on an 11-point scale (-5 = very bad to +5 = very good) to the question How are you feeling right now? Mean pre- and post-workout scores will be calculated for each session [22].  Heart Rate  Participants will be fitted with Polar H7 heart rate monitors during the training sessions, which will be connected to a central iPad application. The mean heart rate for the entire session and the mean maximum heart rate will be tracked over the study period.  Enjoyment and Adherence  Enjoyment will be assessed by the Physical Activity Enjoyment Scale, which is a valid and reliable tool for evaluating perceived enjoyment [23]. Adherence to the intervention program will be evaluated by attendance frequency and dropout rate.  Physical Fitness  Physical fitness (cardiovascular fitness, muscular strength, and speed-agility) will be assessed using the ALPHA fitness test battery [24]. Briefly, cardiovascular fitness will be assessed by the 20 m shuttle run test; muscular strength will be assessed by the handgrip strength test and standing long jump test; and speed-agility will be assessed by the 4 × 10 m shuttle run test [24]. |  | |
|  | 13 |  |  | |
| Participant timeline | 14 | The sample size is calculated using G*Power 3.1. To elucidate the differences in the executive function tests with a statistical power of 0.9, a conservative effect size of 0.65 based on a previous systematic review with the average effect size calculated regarding the effect of exercise on executive function in children with ADHD [25], a two-tailed alpha level of 0.05, it is determined that 10 participants per group will provide adequate power to detect statistically significant differences. Assuming a 30% loss in the intervention, we will need to approach about 42 eligible participants to achieve the planned sample. |  | |
| Sample size | 15 | A total of 42 children with ADHD will be recruited from local schools. Informed consent will be obtained from the school principal, parents, and study participants before the study begins. |  | |
| Recruitment | 15 | A total of 42 children with ADHD will be recruited from local schools. Informed consent will be obtained from the school principal, parents, and study participants before the study begins. |  | |
| **Methods: Assignment of interventions (for controlled trials)** | | |  |  |
| Allocation: | 16a | Participants will be randomly assigned to the GameHIIT group, the GameSAE group, or a non-treatment control group using a random number-producing algorithm (with a 1:1:1 allocation ratio within each school). |  | |
| Sequence generation | 16b | Equal numbers of boys and girls with similar general intelligence will be included in the three groups. Children taking medication will be equally distributed to the three groups. |  | |
| Allocation concealment mechanism | 16c | A stratified random sampling procedure will be conducted that considers gender, IQ, and medication status. |  | |
| Implementation | 17a | Participants will not be blinded to treatment allocation because of the intervention nature. To avoid contamination between treatment groups, intervention deliverers will be provided with a list of students in the intervention program. Only those on the list can participate in the intervention. |  | |
| Blinding (masking) | 17b | N/A. |  | |
|  | 17b | N/A. |  | |
| **Methods: Data collection, management, and analysis** | | |  |  |
| Data collection methods | 18a | Before (T0) and after (T1) the 8-week intervention period, as well as another 8-week follow up (T2), several different indicators will be recorded, including executive function, cerebral hemodynamic response, weekly PA levels, physical fitness, feeling state, and enjoyment and adherence to the intervention. All assessments will be conducted by trained research staff blinded to group allocation. To ensure the accuracy and consistency of the measurements, a measurement training session and protocol manual, including specific instructions for conducting all assessments, will be provided to the research staff. A senior researcher will be present during all the testing sessions. All physical assessments will be conducted in a sensitive manner (e.g., weight/waist circumference will be measured in a private setting), and the cognitive function tests and questionnaires will be completed under exam-like conditions. Also, participants will be instructed to follow similar diets on the main trial days. Only distilled water will be allowed before the tests in the main trials. |  | |
|  | 18b | N/A, there is no participants recruiting in the current study. |  | |
|  | 19 | All information related to participants will remain confidential and will be identifiable by codes known only to the researcher. Entered data will be stored on a password-protected file and a password-protected computer, while original, anonymized hard copies of the questionnaires will be stored in a locked office until 5 years past publication. Only PI, Co-I and his/her research assistants can access the data. |  | |
| Data management | 20a | Statistical analyses of the primary and secondary outcomes will be conducted with the IBM SPSS Statistic for Windows, Version 20.0 (2010 SPSS Inc., IBM Company Armonk, NY). Intervention effects for the primary and secondary outcomes will be examined by two-way (trial × time) analysis covariance (ANCOVA), including group as a fixed factor, pre-post intervention difference (change) as the dependent variable, and age, attendance, sports skill, and ADHD symptoms as covariates. Effect sizes (ES) will be presented as partial eta squared values (ŋ2). Pairwise comparison will be performed (post-hoc) with Bonferroni correction, with ES presented as Cohen’s d. All data will be presented as mean ± SD, and significance will be set as p < 0.05 for all data analysis. |  | |
| Statistical methods | 20b | N/A, no datasets were generated or analysed during the current study. All relevant data from this study will be made available upon study completion. |  | |
|  | 20c | N/A, no additional analyses was generated during the current study. All relevant data from this study will be made available upon study completion. |  | |
|  | 20c | N/A, no analysis population were generated or analysed during the current study. All relevant data from this study will be made available upon study completion. |  | |
| **Methods: Monitoring** | | | 21a | A DMC is not needed, no datasets were generated or analysed during the current study. All relevant data from this study will be made available upon study completion. |
| Data monitoring | 21b | N/A, no datasets were generated or analysed during the current study. All relevant data from this study will be made available upon study completion. |  | |
|  | 22 | N/A, the protocol does not report results |  | |
| Harms | 23 | N/A, no intervention in the current protocol study and no harm may appear through intervention in this study. |  | |
| Confident | 23 | N/A, the current protocol study does not collect data at this moment. |  | |
| Ethics and dissemination | | |  | Human research ethics approval will be sought from the Human Research Ethics Committee of the University. |
| Research ethics  approval | 24 | The ethical approval is given by Human Research Ethics Committee (HREC) at Education University of Hong Kong. |  | |
| Research ethics approval | 25 | The design, conduct, and reporting for the RCT adheres to the guidelines of the Consolidated Standards of Reporting trials (http://www.consort-statement.org/) [13]. |  | |
| Protocol amendments | 26a | Informed consent will be obtained from the school principal, parents, and study participants before the study begins. |  | |
| Consent or assent | 26b | Informed consent will be obtained from the school principal, parents, and study participants before the study begins. Human research ethics approval has been sought from the Human Research Ethics Committee of the University (Ref. no. A2018-2019-0098). |  | |
|  | 27 | All study-related data will be securely stored. All participant data will be kept in sealed file folders with restricted access. To ensure participant privacy, all reports, data collecting, procedure, and administrative forms would be kept in a protected file with restricted access. |  | |
| Confidentiality | 28 | There is no conflict of interests that should be disclosed here. |  | |
| Declaration of interests | 29 | All principal investigators will be given access to the cleaned data sets. Project data sets will be housed on the file transfer protocol site created for the study, and all data sets will be password protected. |  | |
| Access to data | 30 | N/A, there were no application of the intervention in the current study, thus no one may suffer harm from trial participation. |  | |
| Ancillary and post-trial care | 31a | N/A, no results output in the current study. |  | |
| Dissemination policy | 31b | The authors made contributions to the conception or design of the work; the acquisition, analysis, or interpretation of data for the work; and the drafting and the work or revising it critically for important intellectual content. The authors in the study made final approval of the version to be published and had an agreement to be accountable for all aspects of the work in ensuring that questions related to the accuracy or integrity of any part of the work are appropriately investigated and resolved. |  | |
|  | 31c | The future results of the study may be presented in the form of a journal article or a conference presentation or an online web-based report, and personal privacy would be insured without presenting any private information in any form of the presentations and publications. |  | |
|  |  |  |  | |
| Appendices | 32 | Model consent form and other related documentation given to participants and authorised surrogates |  | |
| Informed consent materials | 33 | N/A, no participants recruited in the current study. |  | |
| Biological specimens |  | N/A, no biological specimens applied in his study. |  | |

*It is strongly recommended that this checklist be read in conjunction with the SPIRIT 2013 Explanation & Elaboration for important clarification on the items. Amendments to the protocol should be tracked and dated. The SPIRIT checklist is copyrighted by the SPIRIT Group under the Creative Commons “[Attribution-NonCommercial-NoDerivs 3.0 Unported](http://www.creativecommons.org/licenses/by-nc-nd/3.0/)” license.

**References:**

1. Ng QX, Ho CYX, Chan HW, Yong BZJ, Yeo W. Managing childhood and adolescent attention-deficit/hyperactivity disorder (ADHD) with exercise: A systematic review. Complement Ther Med. 2017;**34**:123-128.
2. Neudecker C, Mewes N, Reimers AK, Woll A. Exercise interventions in children and adolescents with ADHD: A systematic review. J Atten Disord. 2019;**23**(4):307-324. Doi: 10.1177/1087054715584053.
3. Den Heijer AE, Groen Y, Tucha L, Fuermaier AB, et al. Sweat it out? The effects of physical exercise on cognition and behavior in children and adults with ADHD: a systematic literature review. J Neural Transm. 2017;**124**(1):3-26.
4. Cornelius C, Fedewa AL, Ahn S. The effect of physical activity on children with ADHD: A quantitative review of the literature. J Appl Sch Psychol. 2017;**33**(2):136-170.
5. Costigan SA, Eather N, Plotnikoff RC, Taaffe DR, Lubans DR. High-intensity interval training for improving health-related fitness in adolescents: a systematic review and meta-analysis. Br J Sports Med. 2015;**49**(19):1253-1261. Doi: 10.1136/bjsports-2014-094490.
6. Logan GR, Harris N, Duncan S, Schofield G. A review of adolescent high-intensity interval training. Sports Med. 2014;**44**(8):1071-1085. Doi: 10.1007/s40279-014-0187-5.
7. Verburgh L, Königs M, Scherder EJA, Oosterlaan J. Physical exercise and executive functions in preadolescent children, adolescents and young adults: a meta-analysis. Br J Sports Med. 2014;**48**(12):973.
8. Álvarez-Bueno C, Pesce C, Cavero-Redondo I, Sánchez-López M, Martínez-Hortelano JA, Martínez-Vizcaíno V. The effect of physical activity interventions on children’s cognition and metacognition: a systematic review and meta-analysis. J Am Acad Child Adolesc Psychiatry. 2017;**56**(9):729-738. Doi: 10.1016/j.jaac.2017.06.012.
9. Ma JK, Le Mare L, Gurd BJ. Four minutes of in-class high-intensity interval activity improves selective attention in 9- to 11-year olds. Appl Physiol Nutr Metab. 2015;**40**(3):238-244. Doi: 10.1139/apnm-2014-0309.
10. Ma JK, Le Mare L, Gurd BJ. Classroom-based high-intensity interval activity improves off-task behaviour in primary school students. Appl Physiol Nutr Metab. 2014;**39**(12):1332-1337. Doi: 10.1139/apnm-2014-0125.
11. Costigan SA, Eather N, Plotnikoff RC, Hillman CH, Lubans DR. High-intensity interval training for cognitive and mental health in adolescents. Med Sci Sports Exerc. 2016;**48**(10):1985-1993. Doi: 10.1249/MSS.0000000000000993.
12. Meßler CF, Holmberg H, Sperlich B. Multimodal therapy involving high-intensity interval training improves the physical fitness, motor skills, social behavior, and quality of life of boys with ADHD: a randomized controlled study. J Atten Disord. 2016:1505980664.
13. Moher D, Hopewell S, Schulz KF, Montori V, et al. CONSORT 2010 explanation and elaboration: updated guidelines for reporting parallel group randomised trials. BMJ. 2010;**340**:c869.
14. Chang Y, Hung C, Huang C, Hatfield BD, Hung T. Effects of an aquatic exercise program on inhibitory control in children with ADHD: a preliminary study. Arch Clin Neuropsychol. 2014;**29**(3):217-223.
15. Choi JW, Han DH, Kang KD, Jung HY, Renshaw PF. Aerobic exercise and attention deficit hyperactivity disorder: brain research. Med Sci Sports Exerc. 2015;**47**(1):33.
16. Pan C, Tsai C, Chu C, Sung M, Huang C, Ma W. Effects of physical exercise intervention on motor skills and executive functions in children with ADHD: A pilot study. J Atten Disord. 2019;**23**(4):384-397. Doi: 10.1177/1087054715569282.
17. Cooper SB, Bandelow S, Nute ML, Morris JG, Nevill ME. The effects of a mid-morning bout of exercise on adolescents' cognitive function. Ment Health Physic Act. 2012;**5**(2):183-190. Doi: 10.1016/j.mhpa.2012.10.002.
18. Maki A, Yamashita Y, Ito Y, Watanabe E, Mayanagi Y, Koizumi H. Spatial and temporal analysis of human motor activity using noninvasive NIR topography. Med Phys. 1995;**22**(12):1997-2005. Doi: 10.1118/1.597496.
19. Purpura DJ, Lonigan CJ. Conners’ teacher rating scale for preschool children: A revised, brief, age-specific measure. J Clin Child Adolesc Psychol. 2009;**38**(2):263-272. Doi: 10.1080/15374410802698446.
20. Brites C, Salgado-Azoni CA, Ferreira TL, Lima RF, Ciasca SM. Development and applications of the SWAN rating scale for assessment of attention deficit hyperactivity disorder: A literature review. Braz J Med Biol Res. 2015;**48**(11):965-972. Doi: 10.1590/1414-431X20154528.
21. Moore JB, Hanes JJ, Barbeau P, Gutin B, Trevino RP, Yin Z. Validation of the Physical Activity Questionnaire for Older Children in children of different races. Pediatr Exerc Sci. 2007;**19**(1):6-19.0
22. Rejeski WJ, Best DL, Griffith P, Kenney E. Sex-role orientation and the responses of men to exercise stress. Res Q Exerc Sport. 1987;**58**(3):260-264.
23. Kendzierski D, DeCarlo KJ. Physical activity enjoyment scale: Two validation studies. J Sport Exerc Psychol. 1991;**13**(1):50-64. Doi: 10.1123/jsep.13.1.50.
24. Ruiz JR, Castro-Pinero J, Espana-Romero V, Artero EG, et al. Field-based fitness assessment in young people: the ALPHA health-related fitness test battery for children and adolescents. Br J Sports Med. 2011;**45**(6):518-524. Doi: 10.1136/bjsm.2010.075341.
25. Cornelius C, Fedewa AL, Ahn S. The effect of physical activity on children with ADHD: A quantitative review of the literature. J Appl Sch Psychol. 2017;**33**(2):136-170.
26. Chang Y, Liu S, Yu H, Lee Y. Effect of acute exercise on executive function in children with attention deficit hyperactivity disorder. Arch Clin Neuropsychol. 2012;**27**(2):225-237.
